# Prophylactic administration of parenteral steroids for preventing airway complications after extubation in adults: meta-analysis of randomised placebo controlled trials

**DOI:** 10.1136/bmj.a1841

**Published:** 2008-10-20

**Authors:** Tao Fan, Gang Wang, Bing Mao, Zeyu Xiong, Yu Zhang, Xuemei Liu, Lei Wang, Sai Yang

**Affiliations:** 1 Pneumology Group, Department of Integrated Traditional Chinese and Western Medicine, West China Hospital, Sichuan University, Chengdu 610041, China; 2 Division of Pulmonary, Allergy and Critical Care Medicine, Department of Medicine, University of Pittsburgh Medical Center, Pittsburgh, PA 15213, USA; 3 Department of Intensive Care Medicine, First People’s Hospital of Chengdu City, Chengdu 610041, China; 4 Chinese Cochrane Centre, West China Hospital, Sichuan University, Chengdu; 5 Department of Respiratory and Critical Care Medicine, West China Hospital, Sichuan University, Chengdu

## Abstract

**Objective** To determine whether steroids are effective in preventing laryngeal oedema after extubation and reducing the need for subsequent reintubation in critically ill adults.

**Design** Meta-analysis.

**Data sources** PubMed, Cochrane Controlled Trials Register, Web of Science, and Embase with no limitation on language, study year, or publication status.

**Selection criteria** Randomised placebo controlled trials in which parenteral steroids were compared with placebo for preventing complications after extubation in adults.

**Review methods** Search, application of inclusion and exclusion criteria, data extraction, and assessment of methodological quality, independently performed in duplicate. Odds ratios with 95% confidence intervals, risk difference, and number needed to treat were calculated and pooled.

**Main outcome measures** Primary outcome: laryngeal oedema after extubation. Secondary outcome: subsequent reintubation because of laryngeal oedema.

**Results** Six trials (n=1923) were identified. Compared with placebo, steroids given before planned extubation decreased the odds ratio for laryngeal oedema (0.38, 95% confidence interval 0.17 to 0.85) and subsequent reintubation (0.29, 0.15 to 0.58), corresponding with a risk difference of −0.10 (−0.12 to −0.07; number needed to treat 10) and −0.02 (−0.04 to −0.01; 50), respectively. Subgroup analyses indicated that a multidose regimen of steroids had marked positive effects on the occurrence of laryngeal oedema (0.14; 0.08 to 0.23) and on the rate of subsequent reintubation (0.19; 0.07 to 0.50), with a risk difference of −0.19 (−0.24 to −0.15; 5) and −0.04 (−0.07 to −0.02; 25). In single doses there was only a trend towards benefit, with the confidence interval including 1. Side effects related to steroids were not found.

**Conclusion** Prophylactic administration of steroids in multidose regimens before planned extubation reduces the incidence of laryngeal oedema after extubation and the consequent reintubation rate in adults, with few adverse events.

## Introduction

Critically ill patients and those undergoing surgery routinely undergo endotracheal intubation to facilitate mechanical ventilation in the intensive care unit and operating room.^1^ As these patients recover, respiratory support is gradually reduced until the patient can breathe unaided and the endotracheal tube can be removed. Although extubation is generally uneventful, in some patients mechanical irritation by the endotracheal tube causes substantial laryngeal oedema,^2^ despite use of a high volume and low pressure cuff^3^ ^w3^ or laryngeal ultrasound monitoring.^4^ Laryngeal oedema, one of the most common complications, can result in stridor and dyspnoea and need for reintubation.^3^ Such complications, particularly reintubation, might lead to a prolonged stay in intensive care, additional costs, potential morbidity, and mortality.^5^ Any intervention that increases the chances of successful extubation is therefore of great interest.

To avoid airway complications, patients are often given steroids before extubation. Prophylactic steroids substantially reduce the incidence of stridor after extubation in children and tend to decrease the rate of reintubation and stridor in neonates,^6^ ^7^ ^8^ ^9^ but the findings might not be applicable to adults because of differences in anatomy of the upper airway and the approach to airway management. The evidence to support this approach in adults is therefore limited or controversial^6^ ^7^ ^10^ because of the limited number of randomised trials. Previous meta-analyses based on trials in adults up to 2007 yielded inconclusive or negative results and lack reliability because of small sample sizes.^6^ ^7^ ^8^ Furthermore, there have been no subgroup analyses by sex or number of doses or in high risk patients or examining the interval between administration of steroids and extubation.^w4 w5^

We carried out an updated meta-analysis to determine whether steroids are effective in preventing laryngeal oedema after extubation in adults and whether they reduce the need for subsequent reintubation and to examine any reported side effects.

## Methods

### Search strategy and selection criteria

We searched electronic databases including PubMed (1966 to June 2008), CENTRAL (Cochrane Controlled Trials Register; issue 2, 2008), Web of Science (1994 to June 2008), and Embase (1984 to June 2008), adopting the search strategy combining the terms “(anti-inflammatory agents OR glucocorticoid* OR corticosteroid* OR steroid* OR dexamet* OR hydrocort* OR Predniso* OR beclomet* OR methylprednisolone) AND (intubation, intratracheal OR airway obstruction OR laryngeal oedema OR stridor OR extubation)” with the high sensitive searching filter provided by the Cochrane Library.^11^ We identified all clinical trials on steroids and complications after extubation and searched reference lists of review articles and included studies to identify other potentially eligible studies. There was no limitation on language, year of publication, or publication status.

Trials were included if they were randomised placebo controlled trials comparing the prophylactic administration of steroids versus placebo before planned extubation in adults, with adequately reported data on either the occurrence of laryngeal oedema after extubation or the rate of consequent reintubation. After exclusion of duplicates, TF and GW reviewed the full text of all citations with titles and abstracts that seemed to fit the criteria for inclusion. Citations that were clearly not relevant or were not randomised controlled trials were not reviewed in full. The numbers of citations rejected and the reasons for rejection were tracked.

### Data extraction and quality assessment

From each article we extracted details of authors, year of publication, geographical location of the study, study population, sex, sample size, time constraints for investigation after extubation, dropouts or withdrawals, interventions, outcomes, adverse events, and intention to treat analysis.

The two reviewers independently assessed allocation concealment and likelihood of bias to determine methodological quality of the included trials. The allocation concealment was ranked as adequate, uncertain, or clearly inadequate; and the likelihood of bias was scored on a Jadad 5 point scale, which contains two questions each on randomisation and masking and one question on the reporting of dropouts and withdrawals.^12^ Any disagreement between reviewers was resolved by consensus.

### Primary and second outcomes

In intubated patients direct laryngoscopic visualisation, the ideal method for diagnosing laryngeal oedema, requires movement of the endotracheal tube, which might be unsafe in unstable critically ill patients and thus is not routinely applied in clinical practice. After extubation, stridor or dyspnoea—an audible high pitched inspiratory wheeze caused by turbulent airflow through narrowed airways—is generally accepted as a clinical indication of laryngeal oedema.^w2^ We therefore defined minor laryngeal oedema as stridor and dyspnoea after extubation and major laryngeal oedema as severe respiratory distress resulting in tracheal reintubation secondary to upper airway obstruction. Our primary outcome in this meta-analysis was laryngeal oedema after extubation, with subsequent reintubation caused by laryngeal oedema as the secondary outcome.

### Statistical analysis

We treated our two outcomes as dichotomous variables and reported odds ratios with 95% confidence intervals. We examined heterogeneity with the Q statistic (P<0.1, considered significant). We used a random effects model if the Q statistic was significant, otherwise we used a fixed effects model. We carried out subgroup analysis to assess the source of heterogeneity and assessed the presence of publication bias visually with a funnel plot. Differences in risk and number needed to treat were calculated to assess clinical significance. In addition, we calculated the power to confirm the reliability of the analyses.

## Results

### Trials included

Figure 1 shows details of study identification, inclusion, and exclusion[Fig fig1]. Our search strategy initially yielded 1500 citations. Of these, we included six unique studies with 1923 participants in this meta-analysis.^w1-w6^

**Figure fig1:**
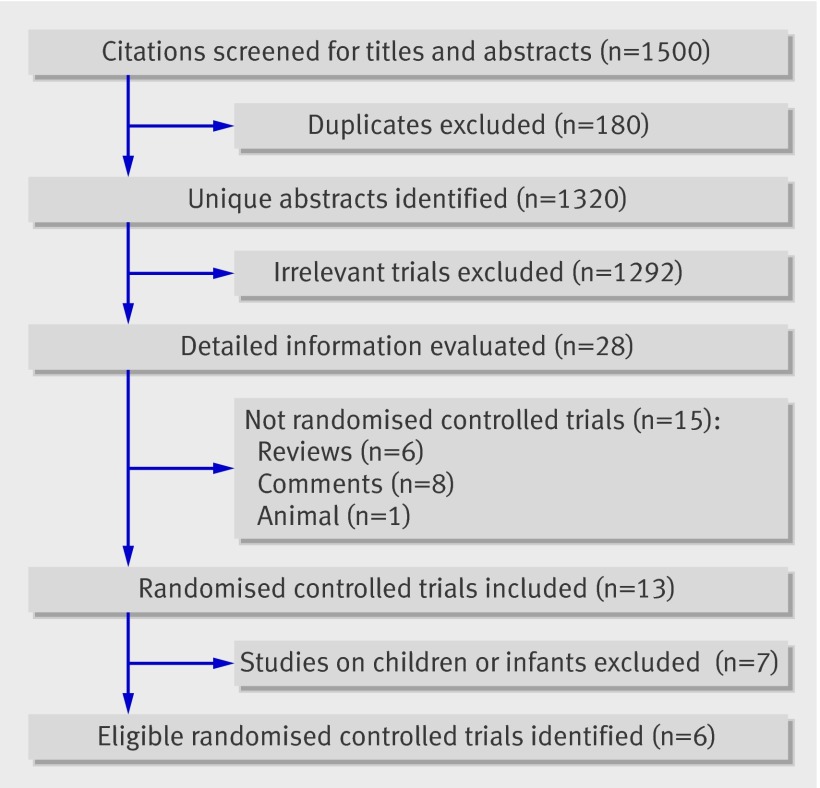
**Fig 1** Flow of study identification, inclusion, exclusion

Tables 1 and 2 show the characteristics of the included studies.[Table tbl1] [Table tbl2] All trials were undertaken in Europe^w1 w2 w5^ and Asia^w3 w4 w6^ in the past 30 years, and three^w4-w6^ were published after 2000. Two trials were multicentre studies.^ w2 w5^ Three of them reported negative results,^w1-w3^ and the others reported positive results.^w4-w6^ Most of the included studies were of high quality (Jadad score ≥3) and clearly reported allocation concealment, except that of Gaussorgues et al (Jadad score 1), which gave no details of randomisation, blinding, or allocation concealment.^w1^ Intention to treat analysis was rarely reported.

**Table 1 tbl1:** Quality assessment of included randomised placebo controlled trials on parenteral steroids for preventing complications in adults after extubation

Reference	Double blinding	Intention to treat	Allocation concealment	Jadad score (0-5)	Adverse events
Gaussorgues 1987^w1^	No	No	Uncertain	1	No
Darmon 1992^w2^	Yes	No	Adequate	5	No
Ho 1996^w3^	Yes	No	Adequate	4	No
Cheng 2006^w4^	Yes	No	Adequate	4	Yes
Francois 2007^w5^	Yes	Yes	Adequate	5	Yes
Lee 2007^w6^	Yes	No	Adequate	5	Yes

**Table 2 tbl2:** Characteristics of included randomised placebo controlled trials on parenteral steroids for preventing complications after extubation in adults

	Steroids	Placebo
**Gaussorgues 1987^w1^ (France and multicentre)**		
Study population	Pneumonia, haemodynamic instability, postoperative, neurological	
Time constraint*	48 hours	
Mean (SD) age (years)	55 (26)	53 (23)
Sample size (% female)	138 (33.3)	138 (36.2)
Intervention in steroid group	40 mg IV and 40 mg IM methylprednisolone 30 min before extubation	—
Laryngeal oedema	4	2
Reintubation	2	0
**Darmon 1992^w2^ (France and multicentre)**		
Study population	Haemodynamic instability, neurological, postoperative	
Time constraint*	24 hours	
Mean (SD) age (years)	49 (18.8) intubated for <36 h; 55.4 (17.9) intubated for >36 h	47.7 (19.4) intubated for <36 h; 59.4 (19.8) intubated for >36 h
Sample size (% female)	348 (39.7)	352 (44.6)
Intervention in steroid group	8 mg IV dexamethasone 1 h before extubation	—
Laryngeal oedema	11	17
Reintubation	2	5
**Ho 1996^w3^ (Taiwan)**		
Study population	Haemodynamic instability, neurological, postoperative, trauma	
Time constraint*	24 hours	
Mean (SD) age (years)	61 (14)	64 (18)
Sample size (% female)	39 (25.6)	38 (21.1)
Intervention in steroid group	100 mg IV hydrocortisone 1 h before extubation	—
Laryngeal oedema	7	10
Reintubation	0	1
**Cheng 2006^w4^ (Taiwan)**		
Study population	Medical, surgical	
Time constraint*	48 hours	
Mean (SD) age (years)	1 injection: 63 (16); 4 injections: 67 (18)	68 (16)
Sample size (% female)	85 (1 injection: n=43 (65.1); 4 injections: n=42 (54.8))	43 (65.1)
Intervention in steroid group	Over 24 h, 40 mg IV infusion methylprednisolone every 6 h (total 160 mg) in 4 injections group; one infusion of methylprednisolone followed by three injections of normal saline every 6 h (total 40 mg) in 1 injection group	—
Laryngeal oedema	5 had 1 injection; 3 had 4 injections	13
Reintubation	2 had 1 injection; 3 had 4 injections	8
**Francois 2007^w5^ (France and multicentre)**		
Study population	Medical, surgical, and trauma (time constraint 24 h*)	
Time constraint*	24 hours	
Mean (range) age (years)	65 (46-75)	66 (48-74)
Sample size (% female)	380 (37.0)	381 (35.0)
Intervention in steroid group	20 mg methylprednisolone initiated 12 h before planned extubation and continued every 4 h with last injection removal (total 80 mg)	—
Laryngeal oedema	11	76
Reintubation	1	14
**Lee 2007^w6^ (Taiwan)**		
Study population	Pneumonia, sepsis, heart failure, acute respiratory distress syndrome, COPD (time constraint 48 h*)	
Time constraint*	48 hours	
Mean (SD) age (years)	72.4 (14.7)	72.7 (13.8)
Sample size (% female)	40 (85.0)	40 (88.0)
Intervention in steroid group	5 mg IV dexamethasone every 6 h, total of four doses with last injection 24 h before extubation	—
Laryngeal oedema	4	11
Reintubation	1	2

*Time constraints for investigation after extubation.

All participants in the six trials were adults who were tracheally intubated for at least 24 hours and were monitored for at least 24 hours after extubation. Two trials mentioned supportive treatments for laryngeal oedema after extubation, such as non-invasive positive pressure ventilation (bi-level positive airway pressure) and the inhalation of racemic adrenaline (epinephrine).^w4 w6^ In these studies patients were usually given intravenous steroids sometime before selected extubation but the steroids used and doses varied (table 2).[Table tbl2]

### Outcomes for meta-analysis 

Table 2 summarises individual study data for the two outcomes.[Table tbl2] In the individual studies, most of the confidence intervals for the odds ratios for laryngeal oedema and reintubation include 1.0, whereas meta-analysis indicated that prophylactic steroids before planned extubation decreased the likelihood of laryngeal oedema after extubation (odds ratio 0.38; 95% confidence interval 0.17 to 0.85) and subsequent reintubation (0.29, 0.15 to 0.58) (figs 2 and 3) [Fig fig3], corresponding with a risk difference of −0.10 (−0.12 to −0.07; number needed to treat 10) and −0.02 (−0.04 to −0.01; 50), respectively. The Q test, however, showed statistical heterogeneity in the occurrence of laryngeal oedema (χ^2^=19.48*, *P*=*0.002, I^2^=74.3%) but not in the rate of reintubation (χ^2^=5.76*, *P*=*0.33, I^2^=13.2%). Reanalyses with a random effects model still suggested that the regimen reduced the occurrence of laryngeal oedema (0.38, 0.17 to 0.85), corresponding with a risk difference of −0.10 (−0.20 to 0.00; 10). The funnel plot showed apparent asymmetry in laryngeal oedema and reintubation.

**Figure fig2:**
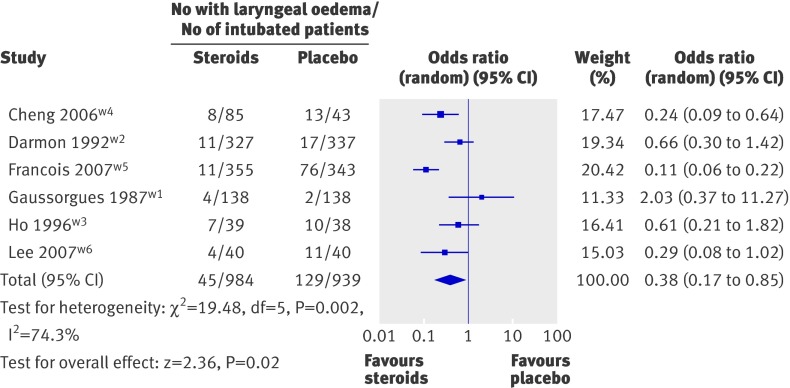
**Fig 2** Effect of steroids on laryngeal oedema after extubation

**Figure fig3:**
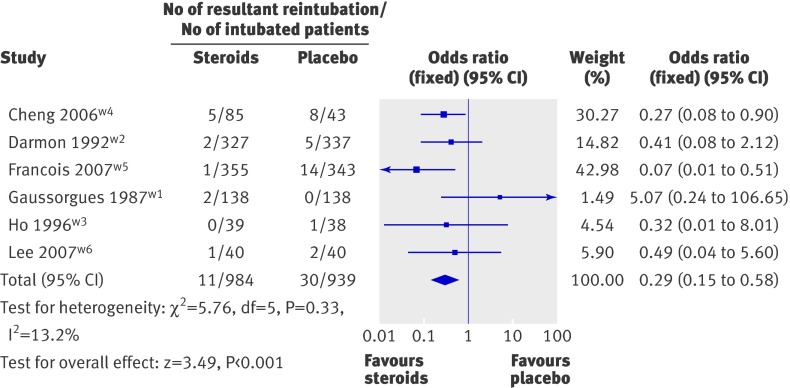
**Fig 3** Effect of steroids on reintubation after extubation

### Subgroup and sensitivity analysis

Table 3 shows the results of subgroup and sensitivity analyses. The total incidence of laryngeal oedema after extubation and subsequent reintubation was 9.0% and 2.1%, whereas in the subgroup with a multidose regimen the figures were 13.7% and 3.4%. In subgroup analyses of different regimens, multiple intravenous steroids had a marked positive effect on the occurrence of laryngeal oedema (odds ratio 0.14, 0.08 to 0.23) and the rate of subsequent reintubation (0.19, 0.07 to 0.50), corresponding with a risk difference of −0.19 (−0.24 to −0.15; number needed to treat 5) and −0.04 (−0.07 to −0.02; 25), but there was only a trend towards benefit with single doses (data not shown). We also carried out subgroup analyses on the relation between dose of steroid and its effect on laryngeal oedema after extubation and reintubation. The included trials used various steroids—such as methylprednisolone, dexamethasone, and hydrocortisone. Because of the different anti-inflammation effects, we converted all doses of steroids to the equivalent dose of methylprednisolone.^13^ There was no significant difference between doses equivalent to 20 mg (odds ratio 0.61 for laryngeal oedema, 0.32 for reintubation) and 40 mg (0.61, 0.47) methylprednisolone, with all confidence intervals including 1 in the single dose regimen. In the multiple dose regimen, however, the effect of steroids equivalent to 160 mg methylprednisolone (0.18, 0.34) was much better than that of 100 mg (0.29, 0.49). In addition, the effect of steroids equivalent to 80 mg methylprednisolone (0.11, 0.07) was the best compared with the steroid doses of 100 mg and 160 mg, but this could have been because that study did not include high risk patients and had a large sample size.^w5^ Exclusion of the low quality study^w1^ (Jadad score <3) and the heaviest weight trial^w5^ did not change the positive direction of our results.

**Table 3 tbl3:** Subgroup and sensitivity analysis of occurrence of laryngeal oedema after extubation and subsequent reintubation

Included trials	Laryngeal oedema						Reintubation				
	No of studies	Steroids	Placebo	Odds ratio (95% CI)	Power		No of studies	Steroids	Placebo	Odds ratio (95% CI)	Power
All studies^w1-w6^	6	45/984	129/939	0.38 (0.17 to 0.85)*	1.00		6	11/984	30/939	0.29 (0.15 to 0.58)	0.93
Excluding study with Jadad score <3^w1^	5	41/846	127/801	0.31 (0.14 to 0.67)*	1.00		5	9/846	30/801	0.22 (0.10 to 0.47)	0.97
Excluding study with heaviest weight^w5^	5	34/629	53/596	0.51 (0.32 to 0.80)	0.77		5	10/629	16/596	0.46 (0.21 to 1.00)	0.38
Inclusion of high risk patients^w4 w6^	2	12/125	24/83	0.26 (0.12 to 0.56)	0.97		2	6/125	10/83	0.31 (0.11 to 0.90)	0.60
**Single dose regimen**											
Overall^w1-4^	4	27/547	42/556	0.61 (0.36 to 1.02)	0.56		4	6/547	14/556	0.45 (0.18 to 1.12)	0.56
Dose equivalent 40 mg†^w1 w2 w4^	3	20/508	32/518	0.61 (0.34 to 1.09)	0.50		3	6/508	13/518	0.47 (0.18 to 1.22)	0.48
Dose equivalent 20 mg†^w3^	1	7/39	10/38	0.61 (0.21 to 1.82)	0.22		1	0/39	1/38	0.32 (0.01 to 8.01)	NA
**Multidose regimen**											
Overall^ w4-6^	3	18/437	100/426	0.14 (0.08 to 0.23)	1.00		3	5/437	24/426	0.19 (0.07 to 0.50)	0.98
Dose equivalent 160 mg†^w4^	1	3/42	13/43	0.18 (0.05 to 0.68)	0.87		1	3/42	8/43	0.34 (0.08 to 1.37)	0.47
Dose equivalent 100 mg†^w6^	1	4/40	11/40	0.29 (0.08 to 1.02)	0.58		1	1/40	2/40	0.49 (0.04 to 5.60)	0.14
Dose equivalent 80 mg†^w5^	1	11/355	76/343	0.11 (0.06 to 0.22)	1.00		1	1/355	14/343	0.07 (0.01 to 0.51)	0.96

NA=not applicable.

*Adoption of random effects model for significant heterogeneity.

†Dose equivalent to dose of methylprednisolone (mg).

Risk factors associated with development of laryngeal oedema after extubation included sex (female),^w5^ high acute physiological and chronic health evaluation score,^3^ low score on the Glasgow coma scale,^w4^ excessive endotracheal tube size^14^ or patient height/tube diameter,^w5^ and a prolonged intubation period.^w5^ Because the presence of an endotracheal tube precludes direct visualisation of the upper airway, recognition of the oedema caused by laryngotracheal injury is often difficult. Upper airway patency, however, can be assessed indirectly in intubated patients by a cuff leak test. A reduced cuff leak volume or cuff leak volume% of tidal volume can predict the occurrence of laryngeal oedema in patients with high risk factors. The use of a cuff leak test before extubation has therefore been recommended as a screening method for laryngeal oedema after extubation.^3^ ^15^ ^16^ Patients with a cuff leak volume <110 ml or 24% of tidal volume (a cut-off point based on the report by Miller and Cole^16^) can be regarded as at high risk. We did subgroup analysis of high risk patients including two trials.^w4 w6^ There were positive effects on the occurrence of laryngeal oedema (0.26, 0.12 to 0.56) and on the rate of subsequent reintubation (0.31, 0.11 to 0.90). We could not carry out a subgroup analysis according to sex as we did not have the required data.

### Adverse events analysis

Three of the studies (n=969) described adverse events,^w4-w6^ but these could not be pooled. In the steroid group, one patient developed septic shock and died 26 hours after extubation, and one developed atelectasis 24 hours after extubation; neither event was considered to be related to the use of steroids. In the placebo group, one patient developed respiratory failure and died 23 hours and 15 minutes after extubation.

## Discussion

### Principal findings

Airway complications after extubation, especially reintubation secondary to laryngeal oedema in adults, are still a problem in intensive care.^17^ In our meta-analysis, the incidence of laryngeal oedema in the placebo group varied from 3% to 30% and the reintubation rates from less than 1% to 5%, possibly because of patients with different risk factors in individual trials. The routine use of steroids in adults before planned extubation, which has been done for decades,^18^ ^19^ is not yet supported by clinical evidence with confidence intervals for odds ratios including 1. By pooling data from individual studies in a systematic review, however, we can gain more test power and add precision to the estimates of effect. The present meta-analysis confirms that, despite various confounding factors, intravenous steroids do decrease the global occurrence of laryngeal oedema after extubation by 62% and the subsequent reintubation by 71%. Multiple dose steroids have a more marked positive effect, leading to a reduction of laryngeal oedema after extubation by 86% and the subsequent reintubation by 81%, but we found no significant differences with single dose regimens. In such strategies, one event of laryngeal oedema after extubation or subsequent reintubation could be avoided by pretreatment with steroids for 10 or 50 patients, whereas with multidose steroids the same benefits can be achieved in five or 25 patients. We also found a possible dose-effect relation between steroid dose and its effect on preventing laryngeal oedema after extubation and reintubation in the multiple dose regimen. Although steroids have several potential adverse events, particularly in patients already at risk of hyperglycaemia and complications of infection, side effects with steroid treatments over 24 hours are minimal.^20^ The included studies found no side effects related to steroids, but adverse events were not often reported. 

### Single *v* multiple dose regimens

Our results confirm the benefit of multidose steroids before planned extubation of adults. It is difficult to predict the risk of laryngeal oedema that will require reintubation. Onset of laryngeal oedema usually occurs within eight hours after extubation^w1 w2 ^21 so administration of steroids immediately after extubation might be too late. Administration of steroids before selected extubation is assumed to protect against or treat mucosal oedema in the glottic region caused by pressure or irritation from the endotracheal tube. Roberts et al compared trials published after 2000 with older studies^22^ and suggested that the lack of clinical benefit observed with earlier studies might have resulted from lower total steroid doses. These authors also pointed out that older studies generally used only a single dose of steroids and suggested that this, compared with the longer duration of treatment in newer studies, might account for the different conclusions. We found possible dose dependent effects in multiple dose regimens but not in single dose regimens. In multiple dose regimens steroids are usually administered at least 12 hours before extubation and repeated almost every plasma half life. This might maintain a high level of anti-inflammatory activity during the period of vulnerability to oedema after extubation.

### Strengths and limitations

As we included high quality randomised controlled trials with strong power and obtained relative narrow confidence intervals, our results are more precise than those from previous studies.^22^ Previous published systematic reviews focusing on the preventive and therapeutic effect of steroids in different age groups showed no clear benefit on the occurrence of airway complications in adults, but our updated results do show such a benefit.^4^ ^5^ ^6^

A recent meta-analysis^10^ found no clear effect of steroids on preventing laryngeal oedema after extubation and reintubation in adults. That study, however, did not include a recent trial with positive results.^w6^ The addition of this small trial, which was not significant in its own right, was sufficient to make the results of our updated meta-analysis significant. The authors of the previous meta-analysis also did not define reintubation as major laryngeal oedema needing tracheal reintubation secondary to upper airway obstruction. This meant that patients^w2^ who needed reintubation during the follow-up period because of clinical deterioration rather than major laryngeal oedema were added to the number of reintubations, especially in the steroid group. The meta-analysis also lacked subgroup analyses of single versus multidose regimens.

Our study also has some limitations. Few of the original studies reported intention to treat analyses, and that reduces the reliability of the data included in this meta-analysis. The use of supportive treatments for laryngeal oedema after extubation in some of the included studies might have reduced the apparent benefit of steroids compared with placebo treatment. The funnel plot shows asymmetry, so we cannot eliminate the possibility of publication bias. Sex is an important risk factor for laryngeal oedema after extubation and subsequent reintubation, but we could not do a subgroup analysis because so few trials reported results separately by sex. Finally, because of heterogeneity we had to use a random effects model, which results in wider confidence intervals and thus a more conservative estimate of treatment effect.

### Conclusion

Our meta-analysis of currently available evidence strongly suggests that prophylactic administration of parenteral steroids in a multidose regimen before planned extubation is effective in reducing the global incidence of laryngeal oedema and subsequent need for reintubation, with few adverse events. Further trials are needed to establish the optimal dose of steroids and the optimal time between the start of treatment and planned extubation.

**What is already known on this topic**

Endotracheal intubation can result in laryngeal oedema; after the tube is removed this might lead to subsequent reintubation
Controversy exists regarding the prophylactic administration of steroids to prevent laryngeal oedema and reintubation in adults

**What this study adds**

Prophylactic administration of steroids before planned extubation reduces the incidence of laryngeal oedema after extubation and the consequent reintubation rate in adults
Multiple dose regimens are more promising than single doses

## Notes

We thank Gu Jun at Technology Consulting Group of the National Library of China for help with literature searching and Guanjian Liu at the Chinese Cochrane Centre for his statistical assistance.

Ethical approval: Not required.

Provenance and peer review: Not commissioned; externally peer reviewed.

## Notes

**Cite this as: ***BMJ* 2008;337:a1841

## References

[1] Stauffer JL, Olson DE, Petty TL. Complications and consequences of endotracheal intubation and tracheotomy: a prospective study of 150 critically ill adult patients. Am J Med 1981;70:65-7. 7457492 10.1016/0002-9343(81)90413-7

[2] Holst B, Berry S, Evans D, Evans R. Laryngeal oedema complicating extubation. Anaesthesia 2007;62:199-200. 10.1111/j.1365-2044.2007.04968.x 17223830

[3] Jaber S, Chanques G, Matecki S, Ramonatxo M, Vergne C, Souche B, et al. Post-extubation stridor in intensive care unit patients. Risk factors evaluation and importance of the cuff-leak test. Intensive Care Med 2003;29:69-74. 12528025 10.1007/s00134-002-1563-4

[4] Ding LW, Wang HC, Wu HD, Chang CJ, Yang PC. Laryngeal ultrasound: a useful method in predicting post-extubation stridor: a pilot study. Eur Respir J 2006;27:384-9. 16452597 10.1183/09031936.06.00029605

[5] Torres A, Gatell JM, Aznar E, el-Ebiary M, Puig de la Bellacasa J, Gonzalez J, et al. Re-intubation increases the risk of nosocomial pneumonia in patients needing mechanical ventilation. Am J Respir Crit Care Med 1995;152:137-41. 7599812 10.1164/ajrccm.152.1.7599812

[6] Meade MO, Guyatt GH, Cook DJ, Sinuff T, Butler R. Trials of corticosteroids to prevent postextubation airway complications. Chest 2001;120(6 suppl):464-8S. 10.1378/chest.120.6_suppl.464s 11742966

[7] Markovitz BP, Randolph AG. Corticosteroids for the prevention and treatment of post-extubation stridor in neonates, children, and adults. Cochrane Database Syst Rev 2000;(2):CD001000. 10.1002/14651858.CD001000 10796576

[8] Davis PG, Henderson-Smart DJ. Intravenous dexamethasone for extubation of newborn infants. Cochrane Database Syst Rev 2001;(4):CD000308. 10.1002/14651858.CD000308 11687075

[9] Markovitz BP, Randolph AG. Corticosteroids for the prevention of reintubation and postextubation stridor in pediatric patients: a meta-analysis. Pediatr Crit Care Med 2002;3:223-6. 12780960 10.1097/00130478-200207000-00003

[10] Markovitz BP, Randolph AG, Khemani RG. Corticosteroids for the prevention and treatment of post-extubation stridor in neonates, children, and adults. Cochrane Database Syst Rev 2008;(2):CD001000. 10.1002/14651858.CD001000.pub2 18425866

[11] Higgins JPT, Green S, eds. Highly sensitive search strategies for identifying reports of randomized controlled trials in Medline. In: Cochrane handbook for systematic reviews of interventions. 4.2.6. Chichester: John Wiley, 2006 (appendix 5b, issue 4, updated Sept 2006).

[12] Jadad AR, Moore RA, Carroll D, Jenkinson C, Reynolds DJ, Gavaghan DJ, et al. Assessing the quality of reports of randomized clinical trials: is blinding necessary? Control Clin Trials 1996;17:1-12. 8721797 10.1016/0197-2456(95)00134-4

[13] Wang G, Wang YJ, Luo FM, Wang L, Jiang LL, Wang L, et al. Effective use of corticosteroids in treatment of plastic bronchitis with hemoptysis in Chinese adults. Acta Pharmocol Sin 2006;27:1206-12. 10.1111/j.1745-7254.2006.00418.x 16923342

[14] Suominen P, Taivainen T, Tuominen N, Voipio V, Wirtavuori K, Hiller A, et al. Optimally fitted tracheal tubes decrease the probability of postextubation adverse events in children undergoing general anesthesia. Paediatr Anaesth 2006;16:641-7. 16719880 10.1111/j.1460-9592.2005.01832.x

[15] Chung YH, Chao TY, Chiu CT, Lin MC. The cuff-leak test is a simple tool to verify severe laryngeal edema in patients undergoing long-term mechanical ventilation. Crit Care Med 2006;34:409-14. 16424722 10.1097/01.ccm.0000198105.65413.85

[16] Miller RL, Cole RP. Association between reduced cuff leak volume and postextubation stridor. Chest 1996;110:1035-40. 8874265 10.1378/chest.110.4.1035

[17] Epstein SK, Ciubotaru RL. Independent effects of etiology of failure and time to reintubation on outcome for patients failing extubation. Am J Respir Crit Care Med 1998;158:489-93. 9700126 10.1164/ajrccm.158.2.9711045

[18] Epstein SK. Preventing postextubation respiratory failure. Crit Care Med 2006;34:1547-8. 16633252 10.1097/01.CCM.0000208325.85450.2F

[19] Kastanos N, Estopa Miro R, Marin Perez A, Xaubet Mir A, Agusti-Vidal A. Laryngotracheal injury due to endotracheal intubation: incidence, evolution, and predisposing factors. A prospective long-term study. Crit Care Med 1983;11:362-7. 6839788 10.1097/00003246-198305000-00009

[20] Hawkins DB, Crockett DM, Shum TK. Corticosteroids in airway management. Otolaryngol Head Neck Surg 1983;91:593-6. 6198612 10.1177/019459988309100601

[21] Mackenzie CF, Shin B, McAslan TC, Blanchard CL, Cowley RA. Severe stridor after prolonged endotracheal intubation using high-volume cuffs. Anesthesiology 1979;50:235-9. 434510 10.1097/00000542-197903000-00015

[22] Roberts RJ, Welch SM, Devlin JW. Corticosteroids for prevention of postextubation laryngeal edema in adults. Ann Pharmacother 2008;42:686-91. 18413685 10.1345/aph.1K655

